# Torsion d’annexe saine et grossesse: à propos d’un cas

**DOI:** 10.11604/pamj.2017.27.197.12250

**Published:** 2017-07-14

**Authors:** Ahmed Guennoun, Yousra Krimou, Nisrine Mamouni, Sanaa Errarhay, Chahrazed Bouchikhi, Abdelaziz Banani

**Affiliations:** 1 Departement de Gynécologie ObstétriqueI, Hôpital Univiersitaire Hassan II, Fez, Maroc

**Keywords:** Torsion d´annexe, annexe saine, complication rare de grossesse, Adnexal torsion, normal adnexa, rare complication of pregnancy

## Abstract

La torsion d'annexe saine lors d’une grossesse est une entité rare. Nous présentons le cas d'une patiente de 22 ans, ayant consulté pour des algies latéro-pelvienne aiguës sur aménorrhée de 2 mois et qui a bénéficié d'une laparotomie exploratrice revenue en faveur d'une torsion sur un ovaire sain en ischémie sévère. Nous avons réalisé une détorsion de l'annexe sans pexie ovarienne. Les suites post-opératoires étaient sans particularité. Un control échographique après 3 semaines en faveur d'une grossesse toujours évolutive. La torsion d'annexe est une urgence à ne pas méconnaître devant toute douleur pelvienne aiguë chez la femme enceinte. Le traitement conservateur est actuellement le gold standard et une prise en charge appropriée est nécessaire pour éviter d'éventuelles complications maternelles et fœtales.

## Abstract (en)

Normal adnexal torsion is rare during pregnancy. We here report the case of a 22-year old patient presenting with acute lateropelvic pain associated with a 2-month history amenorrhea. Exploratory laparotomy showed severe ischemia due to torsion in a normal ovary. The patient underwent adnexal detorsion without ovarian pexy. The postoperative course was uneventful. Ultrasound examination after 3 weeks showed normal pregnancy. Ovarian torsion is an emergency that should not be ignored in pregnant women with acute pelvic pain. Conservative treatment is the gold standard and proper management is necessary to avoid possible maternal and fetal complications.

## Introduction

La torsion d'annexe est une pathologie rare secondaire à la rotation totale ou partielle de l'annexe autour de son axe vasculaire. La survenue d'une torsion d'annexe sur ovaire sain est une situation encore plus rare. L'intérêt de cette situation réside dans sa difficulté diagnostique, et dans le choix de l'attitude thérapeutique à adopter. Nous présentons le cas d'une torsion d'annexe sur ovaire sain survenue au premier trimestre de grossesse.

## Patient et observation

**Figure 1:**
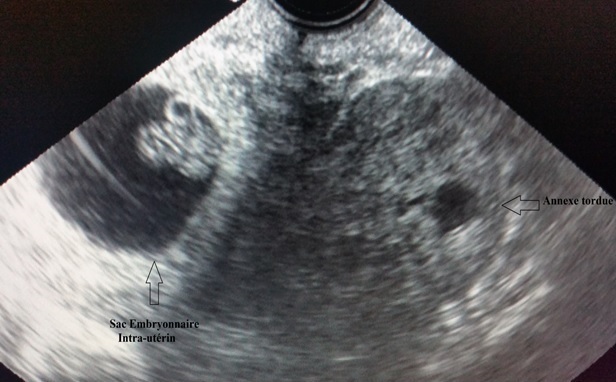
Image échographique endo-vaginale d’un ovaire œdématié sur une annexe tordue

**Figure 2:**
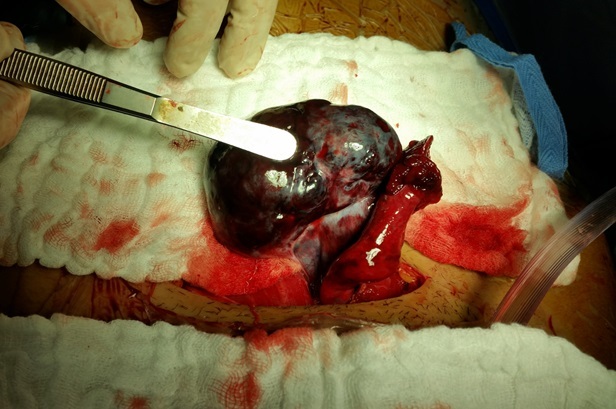
Image peropératoire après détorsion de l’annexe et début de recoloration

Madame N.B âgée de 22 ans, nulligeste. Sans antécédents pathologiques médicaux ou chirurgicaux notables, ayant un cycle régulier avec notion de prise de contraception orale pendant 3 mois arrêtée depuis 3 mois. Elle consulte aux urgences pour des douleurs latéro-pelviennes gauches types de torsion, d'installation aigues évoluant depuis 12 heures, sur une aménorrhée de 8 semaines. L'examen à l'admission trouve une patiente consciente avec un GCS à 15, un EVA à 10. Stable sur le plan hémodynamique et respiratoire: TA 12/7mmhg, FC: 90 bpm, FR 15 C/min, apyrétique T: 37. L'examen abdominal trouve un abdomen souple respire normalement avec présence d'une sensibilité latéro-utérine gauche. A l'examen gynécologique; au spéculum: le col est macroscopiquement normal, pas de saignement provenant de l'endocol. Au Toucher vaginal: l'utérus est augmenté de taille (à 2 TDD de la SP), avec présence d'une sensibilité latéro-utérine gauche. L'échographie (sus-pubienne et endovaginale) est en faveur d'un Sac embryonnaire intra-utérin avec activité cardiaque positive, la longueur cranio-caudale correspondant à 8 semaines. Avec présence en latéro-utérin gauche d'une image échogène légèrement hétérogène avec des zones anéchogènes de 65x45mm sans prise de Doppler faisant évoqué un gros ovaire tordu (Figure 1). La patiente a bénéficié d'une laparotomie de type mini-Pfannenstiel, on a noté à l'exploration la présence d'une fine lame d'épanchement, un utérus augmenté de taille avec annexe droite sans particularité. L'annexe gauche était en ischémie sévère (Figure 2), tordue d'un tour de spire, sans masse kystique individualisable et un ligament utéro-ovarien d'aspect normal. La décision était donc de réaliser une détorsion de l'annexe avec une ponction ovarienne revenue négative, sans ovariopexie. Les suites post-opératoires sont sans particularité avec disparition de la douleur. Un control échographique après 3 semaines trouve une grossesse toujours évolutive.

## Discussion

La torsion d'annexe durant la grossesse est une urgence rare. Son incidence varie de 3 à 5 pour 10000 grossesses [1, 2]. Entre 8 et 28% des torsions surviennent en cours de grossesse [3, 4], majoritairement au premier trimestre mais peuvent être diagnostiquées à tout âge de la grossesse [4]. Habituellement la torsion se produit sur un ovaire pathologique (tumeur maligne ou bénigne). La symptomatologie se caractérise en générale par une douleur pelvienne latérale brutale associée à des nausées et des vomissements. Son diagnostic durant la grossesse est rendu complexe car il nécessite l'élimination des diagnostics différentiels classiques mais aussi ceux pouvant être liés à la grossesse (fausse couche, hématome rétro-placentaire, rupture utérine). De plus, l'examen clinique comme les examens d'imagerie deviennent plus difficile en raison du volume utérin et de l'ascension concomitante de l'ovaire dans la cavité abdominale. La torsiond'annexe peut causer des contractions utérines, entraînant ainsi un risque defausse couche (précoce ou tardive) ou d'accouchement prématuré selon l'âge de grossesse. Le bilan biologique n'est pas spécifique, on peut avoir une hyperleucocytose ou une augmentation de la C-réactive protéine, signant un processus inflammatoire. L'échographie même si elle est pauvre en signes positifs de torsion, elle reste l'examen de référence. Elle permet d'éliminer les diagnostics différentiels, et de recherche les facteurs pouvant favoriser la torsion ainsi que les signes indirects d'ischémie. L'interruption du flux veineux entraîne un œdème réactionnel qui est repérable par l'augmentation du volume ovarien comparativement à l'annexe controlatérale [5, 6]. Par ailleurs, l'augmentation du nombre des follicules corticaux est un aspect non spécifique mais qui a été de multiple fois retrouvé dans le cas de torsion sur ovaire sain. Cet aspect de structure folliculaire homogène et périphérique a été retrouvé dans notre case report. L'utilité du doppler des vaisseaux ovariens reste controversée. Bien que l'absence de signal doppler confirme l'absence du flux sanguin et donc la torsion, l'inverse est faux [7]. L'IRM est une technique d'exploration complémentaire satisfaisante chez la femme enceinte, qui a une plus grande précision que l'échographie [8]. L'association du doppler et de l'IRM permet une meilleure approche diagnostic mais ne doit pas retarder la prise en charge chirurgicale.

La torsion d'annexe est une véritable urgence chirurgicale. Actuellement la cœlioscopie est recommandée pour un âge gestationnel inférieur à 17 SA avec toutefois des consignes de sécurité à respecter: l'utilisation préférentielle d'une open-cœlioscopie, une pression d'insufflation entre 8 et 12 mmHg, une position des trocarts adaptée et enfin une mobilisation douce de l'utérus. Le traitement conservateur ou radical est décidé en fonction de l'aspect de l'annexe 10 minutes après la détorsion [9]: pour les stades 1 et 2 (lésions avec récupération totale ou partielle après détorsion), un traitement conservateur est recommandé, permettant une récupération fonctionnelle dans 90% des cas. Pour un stade 3 (lésions nécrotiques, noires et friables sans récupération après détorsion), l'annexectomie apparaît préférable. Pour d'autres auteurs, la grande capacité de récupération fonctionnelle du tissu ovarien justifie d'être conservateur même devant une annexe de vitalité douteuse [9–13]. La pexie d'ovaire est indispensable quand il existe une malformation des ligaments de l'ovaire, ou une récidive immédiate de la torsion [14].

## Conclusion

Le diagnostic de torsion d'annexe reste difficile, en particularité pendant la grossesse et encore plus en présence d'une annexe saine. En effet, le tableau clinique est peu spécifique, les examens paracliniques sont peu fiables pour poser le diagnostic positif mais gardent leur place afin d'éliminer les différents diagnostics différentiels et rechercher une pathologie annexielle. Le geste opératoire doit être conservateur et consiste en la détorsion de l'annexe, l'ovariopexie ne doit pas être systématique. Le pronostic gravidique est en générale favorable, quelques cas de retard de croissance et d'accouchement prématuré ont été décrits.

## Conflits d’intérêts

Les auteurs ne déclarent aucun conflit d'intérêts.
